# Antidiabetic and Antigout Properties of the Ultrasound-Assisted Extraction of Total Biflavonoids from *Selaginella doederleinii* Revealed by In Vitro and In Silico Studies

**DOI:** 10.3390/antiox13101184

**Published:** 2024-09-30

**Authors:** Qiong Gao, Lei Qiao, Yiru Hou, Hailin Ran, Feng Zhang, Chao Liu, Juxiang Kuang, Shixing Deng, Yongmei Jiang, Gang Wang, Xin Zhang

**Affiliations:** 1 School of Pharmacy, Zunyi Medical University, Zunyi 563000, China; 2 Key Laboratory of Basic Pharmacology of Ministry of Education and Joint International Research Laboratory of Ethnomedicine of Ministry of Education, School of Pharmacy, Zunyi Medical University, Zunyi 563003, China

**Keywords:** *Selaginella doederleinii*, bioflavonoids, enzyme inhibition, molecular docking

## Abstract

In this study, the extraction, purification and metabolic enzyme inhibition potential of *Selaginella doederleinii* were investigated. In order to extract the total biflavonoids from *S. doederleinii* (SDTBs), the optimum extraction process was obtained by optimizing the ultrasonic extraction parameters using response-surface methodology. This resulted in a total biflavonoid content of 22.26 ± 0.35 mg/g. Purification of the *S. doederleinii* extract was carried out using octadecylsilane (ODS), and the transfer rate of the SDTBs was 82.12 ± 3.48% under the optimum purification conditions. We determined the effect of the SDTBs on α-glucosidase (AG), α-amylase and xanthine oxidase (XOD) and found that the SDTBs had an extremely potent inhibitory effect on AG, with an IC_50_ value of 57.46 μg/mL, which was much lower than that of the positive control. Meanwhile, they also showed significant inhibition of XOD and α-amylase, with IC_50_ values of 289.67 μg/mL and 50.85 μg/mL, respectively. In addition, molecular docking studies were carried out to understand the nature of the action of the biflavonoids on AG and XOD. The results showed that robustaflavone had the lowest binding energy to AG (−11.33 kcal/mol) and XOD (−10.21 kcal/mol), while, on the other hand, amentoflavone showed a good binding affinity to AG (−10.40 kcal/mol) and XOD (−9.962 kcal/mol). Moreover, molecular dynamics simulations verified the above results.

## 1. Introduction

*Selaginella doederleinii* (SD) is a perennial herb in the genus *Selaginella* of the family *Selaginellaceae*, and the whole herb is used as medicine [1]. It is mainly distributed in the southwest of China, India, and Thailand [2]. The whole herb has traditionally been used as folk medicine to promote health and treat certain inflammatory [3] and cancerous diseases [4,5]. Previous studies have shown that its active ingredients include flavonoids [6,7,8], lignans [9], alkaloids [10] and polysaccharides [11]. And among them, biflavonoids are the main active ones [12,13,14].

Biflavonoids are naturally occurring dimers made out of individual flavonoid derivatives according to ether oxygen bond condensation or carbon–carbon condensation. They have good antioxidant [15,16,17], hypoglycemic [18], antitumor [19,20], anti-inflammatory [21] and other pharmacological effects. Frota et al. [22] reported the DPPH+ (1,1-diphenyl-2-picrylhydrazyl) radical scavenging ability of amentoflavone (AMF), with an IC_50_ of 5.73 ± 0.08 µg/mL. An in silico study of the antiradical properties of the molecule revealed the best trend in its antiradical mechanisms for the hydrogen atom transfer (HAT) mechanism. According to research by Jeong et al. [23], ginkgetin and AMF inhibit ERK1/2 activation and/or preserve antioxidant enzyme activity, hence shielding HT22 neurons from glutamate-induced oxidative damage. Li et al. [24] reported that AMF and hinokiflavone (HIF) can effectively inhibit the activity of alpha-glucosidase (AG) and can be used as potential hypoglycemic functional foods for diabetes treatment.

Gout and diabetes mellitus are chronic illnesses that are common around the world and have a serious negative impact on human life [25,26]. As a result, they have gained international attention. Several enzymes, such as AG, α-amylase and xanthine oxidase (XOD), play a crucial role in the development of these diseases [27,28]. Therefore, the use of certain substances to inhibit the activity of these enzymes and thereby impede the progression of the diseases in question is an effective strategy within the pharmaceutical industry. However, a lot of these clinical enzyme inhibitors are synthetic artificial substances like allopurinol and acarbose that exhibit certain toxic side effects [29,30]. Finding safer and more potent inhibitors from natural sources is therefore a desirable choice. Flavonoids from foods and medicinal plants have been proven in numerous studies to be prospective inhibitors of different metabolic enzymes [31,32,33]. High-purity flavonoid extraction from SD is an extremely difficult process. The main techniques used to obtain high-purity flavonoids include liquid–liquid extraction and solid-phase extraction (SPE). Richer flavonoid fractions are obtained from raw extract by liquid–liquid extraction with organic solvents [34]. However, the majority of organic solvents—including ethyl acetate—are poisonous, and breathing them in or ingesting them repeatedly can have detrimental effects on human health. Consequently, it is advised to employ safer substitutes for SPE methods, such as using macroporous resins, silica gel, dispersants, polyamide or Sephadex LH-20 [7].

In this study, a method was developed for the determination of the total biflavonoids in *Selaginella doederleinii* (SDTB), and optimization of the process of obtaining the SD crude extract was investigated using ethanol ultrasonic extraction. Response-surface analysis and a one-way experiment were used to adjust the extraction temperature, extraction power, ethanol concentration, ultrasonic duration and liquid–solid ratio. The quality of SD crude extract was examined in terms of its total biflavonoid content, and the SD crude extract was purified by octadecylsilane (ODS). Moreover, the in vitro enzyme inhibitory properties of the purified SD compounds were assessed, and molecular docking was used to study the mechanisms of interaction of the biflavonoids with the enzymes.

## 2. Materials and Methods

### 2.1. Chemicals and Reagents

The herb was extracted in Longyan City, Fujian Province, China, and identified as dried *Selaginella doederleinii* by Associate Prof. Zhang Yujin from the Department of Pharmacognosy, Zunyi Medical University. The control herb for the experiment was purchased from Chengdu Grass Source Kang Biotechnology Co., Chengdu, China, with batch number 380031-202301.

The standards for AMF, robustaflavone (ROF), HIF and Heveaflavone (HEF) were purchased from Shanghai Yuanye Biotechnology Co., Shanghai, China; AG, α-amylase, XOD and xanthine were purchased from Shanghai Yuanye Biotechnology Co., Shanghai, China; ODS and macroporous resin (SP825, FPX-66, NAK-9, NAK-2, HPD-100, D101, AB-8) were purchased from Beijing Huideyi Technology Co., Ltd., Beijing, China; and the DNS reagents were purchased from Solebao Biotechnology Co., Beijing, China. Unless otherwise specified, all the reagents were purchased from Shanghai Yuanye Biotechnology Co., Shanghai, China.

### 2.2. Establishment of Analytical Methods

#### 2.2.1. Determination of Total Biflavonoids (TF) Content

The TF content was determined by ultraviolet spectrophotometry (UV) according to a previous study [35]. AMF was utilized as a standard, with concentrations ranging from 10.82 to 75.74 μg/mL. A calibration curve (y = 0.0642x − 0.1832, R^2^ = 0.9999) was established with a good linear range. The TF content was expressed in milligram equivalents (mg/g) of AMF per gram of SD (dry weight).

#### 2.2.2. Determination of Major Biflavonoids

**Figure 1:**
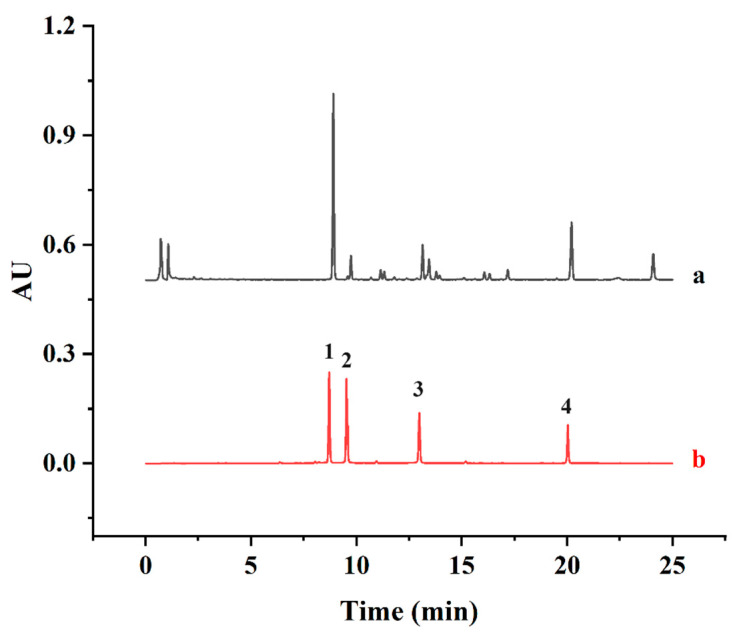
Chromatograms of samples (a) and mixed standards (b) (1 for Amentoflavone (AMF), 2 for Robustaflavone (ROF), 3 for Hinokiflavone (HIF), 4 for Heveaflavone (HEF)).

A UPLC method was developed to investigate the major biflavonoids in SD. Based on the method of Xie [36] with some modifications, the contents of four major biflavonoids, AMF, ROF, HIF and HEF, were simultaneously determined by UPLC and summed up as the SDTB contents. The biflavonoids were separated on a Titank C18 column (1.8 μm, 100 × 2.1 mm). The mobile phases were acetonitrile (A) and 0.1% formic acid in water (B). The gradients for elution were as follows: 0–10 min, 30–50% A; 10–20 min, 50–70% A; and 20–25 min, 70–80% A. Furthermore, the detection wavelength, column temperature, flow rate and injection volume were maintained at 330 nm, 40 °C, 0.3 mL/min and 1 µL, respectively. The content of SDTB was expressed in mg/g. The retention times of the four standards, AMF, ROF, HIF and HEF (8.86, 9.55, 13.01 and 20.06 min, respectively), were used to identify each peak in the UPLC profile of the SDTB extract (Figure 1). The total amount of biflavonoids in the extract was calculated using the following Equation (1):

SDTB content (mg/g) = ((AMF amount + ROF amount + HIF amount + HEF amount)/(Initial sample amount)) × 100% (1)

### 2.3. Optimization of SD Extraction Process

#### 2.3.1. One-Way Experiment

In total, 1.0 g of SD herb powder was weighed precisely, and ethanol was added for ultrasonic extraction. In order to examine how the extraction parameters affected the SDTB extraction rate, the extracted material was filtered and fixed to a specific volume after the extraction process was completed. The extraction factors were designed as the extraction time (20 min, 30 min, 40 min, 50 min, and 60 min), ultrasonic power (200 W, 240 W, 280 W, 320 W, and 360 W), ethanol concentration (50%, 60%, 70%, 80%, and 90%), extraction temperature (30 °C, 40 °C, 50 °C, 60 °C and 70 °C) and liquid–solid ratio (10 mL/g, 15 mL/g, 20 mL/g, 25 mL/g, and 30 mL/g). 

#### 2.3.2. Response-Surface Methodology (RSM) 

The Box–Behnken design (BBD) was utilized to investigate the interactions between the primary variables based on the one-way experiment results. Three significant factors (extraction time (A), extraction power (B), and ethanol concentration (C)) were selected to optimize the extraction process of SDTB using the response surface methodology. The factor level results of Box–Behnken central combinatorial design using design-Expert 13 software are shown in Table 1. 

### 2.4. Purification of SD Extracts

#### 2.4.1. Preparation of the Extract

The extract was prepared under the optimal conditions obtained in “Section 3.1.2” and stored at 4 °C.

#### 2.4.2. Comparison of Different Fillers

After loading the chromatographic column with 5.0 g of treated octadecylsilane (ODS) and macroporous resins (SP825, FPX-66, NAK-9, NAK-2, HPD-100, D101, AB-8), the column was attached to a constant-flow pump (HL-2B, Shanghai Qingpu Husi Instrument Co., Shanghai, China). Methanol was used as the eluent to fill the column with methanol at an appropriate flow rate, and air bubbles were evacuated. In total, 10 mL of the SD extract (1 mg/mL) was added, and 200 mL of 80% methanol was used as the eluent. The flow rate was adjusted to 5 mL/min, and 200 mL of eluate was collected. To find the ideal elution packing, the evaluation criterion used to select candidates was the transfer rate of SDTB (2).

Transfer rate = total biflavonoids content in the elution solution/total biflavonoids content in the sample loading solution × 100% (2)

#### 2.4.3. Effect of Methanol Concentration

Methanol is often used as an elution solvent for flavonoids, and the difference in methanol concentration will directly affect the elution efficiency and the purity of the target product after enrichment [37,38]. In total, 5.0 g of optimal filler was weighed and added to 10 mL of SD extraction solution (1 mg/mL) at a flow rate of 5 mL/min. Total 200 mL of eluate was collected after elution with methanol at concentrations of 60%, 70%, 80%, 90%, and 100%, respectively. The SDTB content was determined, and the transfer rate was calculated to evaluate the optimal elution concentration.

#### 2.4.4. Effect of Elution Flow Rate

One important influencing aspect in the enrichment process is the elution flow rate [39]. A total of 5.0 g of the optimal packing material was weighed, and 10 mL of SD extract (1 mg/mL) was added and eluted with 80% methanol solution. The flow rates were 1 mL/min, 3 mL/min, 5 mL/min, 7 mL/min, and 9 mL/min. Then, 200 mL of the eluate was collected and the amount of SDTB in the eluate was measured and the transfer rate was calculated to determine the optimal elution flow rate.

#### 2.4.5. Effect of Sample Concentration

An excessive loading volume will lead to an overload of the filler, which will affect the purity of the target product; therefore, an appropriate loading volume is necessary [40]. An optimal filler, 5.0 g, was weighed and added to 10 mL of SD extract at concentrations of 0.5 mg/mL, 1.0 mg/mL, 1.5 mg/mL, 2.0 mg/mL, and 2.5 mg/mL. Elution was performed with an 80% methanol solution at a flow rate of 5 mL/min. In total, 200 mL of eluate was collected, and the optimal elution flow rate was determined by measuring the amount of SDTB and calculating the transfer rate.

### 2.5. Enzyme Inhibition Assay of SD Purifications

#### 2.5.1. Inhibitory Activity of α-Glucosidase (AG)

With a small modification, the method of Rodríguez et al. [41] was employed to assess the inhibitory activity of SD extracts against α-glucosidase within a concentration range of 10–150 μg/mL. The IC_50_ values were subsequently computed. The principle is that p-nitrophenyl-α-d-glucopyranoside (PNPG) used as a substrate is converted into yellow p-nitrophenol (PNP) and glucose by α-glucosidase, and PNP exhibiting maximum absorption at 405 nm. The specific procedure is shown in Table 2. 

Acarbose was used as a positive control. The rate of enzyme activity inhibition was calculated using Equation (3):

(3) $$Inhibition\;rate\left(\%\right)=(1-\frac{A_{Sample}-A_{Sample\;Blank}}{A_{Control}})\times 100\%$$

#### 2.5.2. Inhibitory Activity of α-Amylase

Based on the method of Olufolabo [42] with slight modifications, the inhibitory activity of SD extracts within a concentration range of 5–100 μg/mL against α-amylase was determined, and the IC_50_ value was calculated. The principle is that α-amylase can hydrolyze the substrate starch into reducing sugars, which produce a reddish-brown color through the reaction of 3,5-dinitrosalicylic acid with reducing sugars, and the absorbance was measured at 540 nm. The specific steps are shown in Table 3.

Acarbose was used as a positive control. The rate of enzyme activity inhibition was calculated using Equation (3).

#### 2.5.3. Inhibitory Activity of Xanthine Oxidase (XOD)

Following the method of Tang [43] with slight adjustments, the inhibitory activity of SD extracts within a concentration range of 10–400 μg/mL against XOD was determined, and the IC_50_ value was calculated. The specific principle is that xanthine serves as a substrate and XOD catalyzes the conversion of uric acid from xanthine, which can be detected by the absorbance at 290 nm to calculate the XOD inhibitory activity. The specific steps are shown in Table 4. 

Allopurinol was used as a positive control. The rate of enzyme activity inhibition was calculated using Equation (4):

(4) $$Inhibition\;rate\left(\%\right)=(1-\frac{A_{Sample}-A_{Sample\;Blank}}{A_{Control}-A_{Control\;Blank}})\times 100\%$$

### 2.6. Molecular Docking of Oxidative Enzymes

#### 2.6.1. Ligand and Receptor Structure Preparation

**Figure 2:**
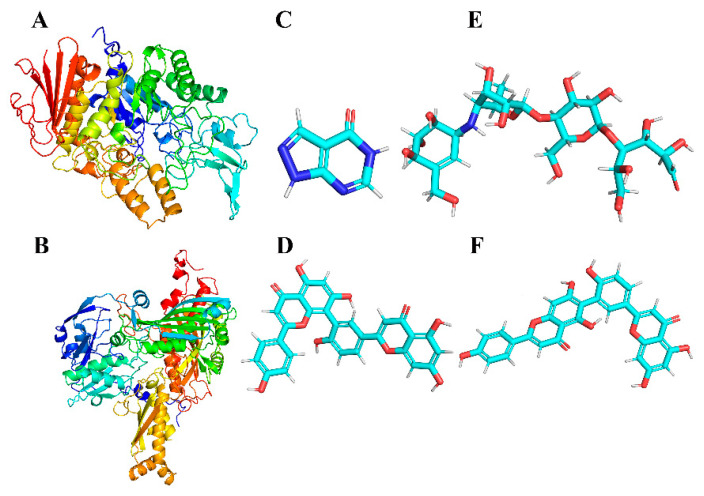
Three-dimensional structures of the proteins (represented by a cartoon model) α-glucosidase (**A**) and XOD (**B**); three-dimensional structures of small-molecule ligands allopurinol (**C**), AMF (**D**), acarbose (**E**) and ROF (**F**).

Protein information for AG (PDB ID:3A4A) and XOD (PDB ID:3NVY) was extracted from the PDB database (https://www.rcsb.org/, accessed on 7 March 2024). The pdb structures were downloaded to serve as the initial structure of the protein receptor and residue complementation was performed. The ligand small molecule in mol format was converted into a 3D molecular structure by OpenBabel.2.3.1 [44] software. The MMFF94 force field was applied to optimize the 3D structures of small molecules. Ultimately, the initial receptor and ligand structures were processed, hydrogenation was performed, charges were calculated and saved, docking atom types were assigned and pdbqt files were generated for docking using AutoDock Tools 1.5.6 [45]. The ligand and receptor structures are shown in Figure 2.

#### 2.6.2. Docking

Docking was performed using AutoDock 4.2 [46], with the center coordinates of the docking box set at the known structural domain of the protein. With the grid point size set to 0.375 Å, all potential amino acid that could bind were included. The default values were applied to the remaining parameters. Docking was executed, and the 10 highest-scoring docked structures were provided for each dock. The lowest-scoring small molecule that located within the structural domain was selected for analysis. Ligand–protein interactions were using Ligplot 2.2.8 [47] software and visualization views were drawn by PyMOL 2.5.0. 

### 2.7. Molecular Dynamic Simulation Method

To further investigate the interactions and stability of the complex, molecular dynamics (MD) simulations were conducted using GROMACS 2022.3 to simulate the protein–ligand complex for 50 ns [48]. The AMBER99SB-ILDN force field was selected to generate the protein topology, while the ligand topology was generated using the GAFF force field through the Amber20 software [49]. A truncated octahedral TIP3P water box was added around the system maintaining a distance of 10 nm, and Na^+^/Cl^−^ ions were included to neutralize the system [50]. Energy minimization was performed using 2500 steps of the steepest descent method followed by 2500 steps of the conjugate gradient method. Subsequently, an NVT ensemble simulation for 100 ps and an NPT equilibrium simulation for 100 ps were conducted out at a constant temperature of 298.15 K [51]. Finally, a 50 ns MD ensemble simulation was performed under periodic boundary conditions, with long-range electrostatic interactions calculated using the Particle Mesh Ewald (PME) method, a non-bonded cutoff distance of 1 nm, a collision frequency of 2 ps, a system pressure of 101.325 kPa and a time step of 2 fs. Trajectories were saved every 10 ps [52].

Using the molecular dynamic simulations, the binding free energy between the protein and the ligand were calculated, offering deeper insights into the mechanism of their interactions. This analysis offers valuable information for drug design and therapeutic development by elucidating the principles governing protein–ligand binding. Additionally, the stable molecular conformations from the final 20 ns of the equilibrated trajectory were extracted. The binding free energy was further calculated using the gmx MMPBSA 1.6.2 tool, which aids in understanding the nature, strength and types of interactions between the protein and the ligand.

### 2.8. Statistical Analysis

In this study, all experiments were conducted in triplicate, and the experimental data are presented as the mean ± standard deviation (±SD). Design Expert 8.0.6 was used to optimize the extraction process, and SPSS 18.0 was used for statistical analysis. A *p*-value of less than 0.05 (*p* < 0.05) was considered statistically significant. Comparisons of the mean ± standard deviation data were performed using the ANOVA followed by Duncan’s multiple range test, which assigns a set of letters. Different letters indicate significant differences between values. Double letters indicate partial differences between the corresponding values.

## 3. Results and Discussion

### 3.1. Analysis of the Extraction Process

#### 3.1.1. One-Way Experiment Analysis

**Figure 3:**
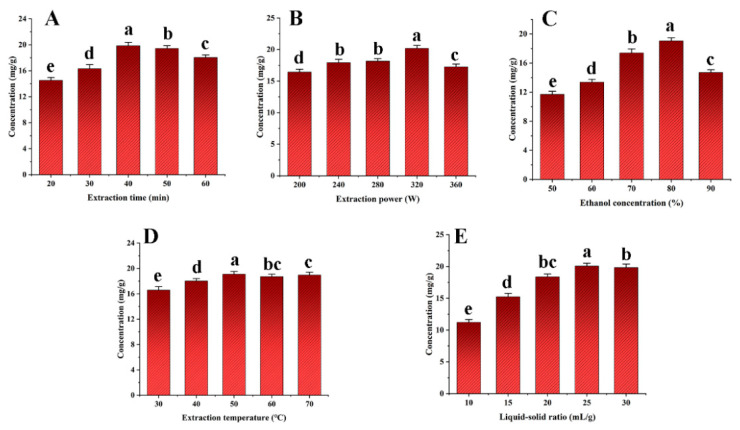
Effects of extraction time (**A**), extraction power (**B**), extraction concentration (**C**), extraction temperature (**D**) and liquid-solid ratio (**E**) on the total biflavonoids from *Selaginella doederleinii*.

Important process variables, including the extraction duration, ultrasonic power, ethanol concentration, extraction temperature, and liquid–solid ratio were carefully examined to increase the SDTB extraction rate. The results are shown in Figure 3. The highest extraction rate of SDTB was achieved when the extraction time, ultrasonic power and ethanol concentration were 40 min, 320 W and 70%, respectively. Extraction time can affect the exudation of natural compounds from the plant, leading to changes in the extraction rate. Therefore sufficient time allows the extraction process to reach equilibrium [53]. Ultrasonic power, through the cavitation effect, which intensifies with increasing power and cavitates the bubbles, influences the extraction rate of herbal medicines [54]. According to Samaram et al. [55], as ultrasonic power increases, hydrodynamic forces rise, tending to break down cell walls and boost yield. However, excessive ultrasonic power may increase the number of bubbles in the solvent during cavitation, reducing the efficiency of the ultrasonic energy transferred into the medium and decrease the yield [56]. Typically, methanol, ethanol, and acetone are suitable for the extraction of biflavonoids from herbs [57]. Different ethanol concentrations can affect the rate of dissolution and extraction of natural products from plants. Therefore, extraction time (30 min, 40 min, 50 min), ultrasonic powers (280 W, 320 W, 360 W), and ethanol concentrations (70%, 80%, 90%) were selected for subsequent studies.

We carried out research on different extraction methods for *Selaginella* plants. For example, Lei et al. [35] extracted *Selaginella moellendorffii* by the ultrasound-assisted natural deep eutectic solvents (NADES) method. The optimal extraction conditions were a 24% water content of NADES, ultrasonic power of 260 W, liquid–solid ratio of 24:1 mL/g and extraction time of 43 min. Under these conditions, 5.72 ± 0.13 mg/g of total flavonoids was extracted, almost three times more than with ionic liquid extraction and conventional techniques. The total flavonoids (TFs) from *Selaginella involvens* Spring were extracted by homogenate-ultrasound-assisted ionic liquid extraction (HUA-ILE) [58]. The extraction rate was 8.48 ± 0.27 mg/g under the optimal extraction conditions. Compared with the conventional methods, the HUA-ILE method showed a four times higher TF content and a 100 times shorter extraction time. This study examined the process parameters using conventional ethanol as the extraction solvent because these novel green solvents are not sufficiently yet developed enough to be used in industrial extraction.

#### 3.1.2. RSM Optimized Extraction Process

One-way experiments, although optimizing the extraction process of the target components to some extent, do not accurately predict the optimal experimental conditions. Therefore, in this study, the RSM was chosen to obtain the optimal process parameters. The results are shown in Table 5 and the analysis of variance (ANOVA) is presented in Table 6. In the ANOVA, the model had an F-value of 40.19, *p* < 0.0001, a coefficient of determination R^2^ of 0.9810 and a correction coefficient R^2^ (Adj) of 0.9566, indicating that the regression equations were well fitted and statistically significant. Furthermore, the experimental predictive model fits the measured values well and can accurately depict the link between the individual components and changes in the response values, as evidenced by the fact that the difference between the model misfit terms is not significant (*p* > 0.05). The quadratic terms of extraction time (A), extraction power (B) and ethanol concentration (C) had highly significant effects on the response values (*p* < 0.05), according to the ANOVA of the model. The order of significance for the effects of each factor on the response values was C > B > A. The response-surface regression model was analyzed using the quadratic response-surface regression method and the following multivariate quadratic. The binary regression equation model for the extraction rate of SDTB (*Y*) was obtained as follows (5):

*Y* = 22.16 + 0.2288 × A − 0.7843 × B + 0.3858 × C − 0.2166 × AB − 0.2202 × AC + 0.9583 × BC − 0.8315 × A^2^ − 1.35 × B^2^ − 2.46 × C^2^ (5)

**Figure 4:**
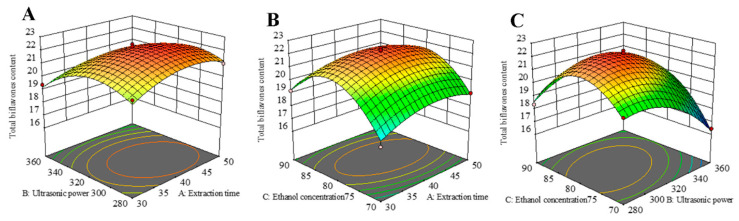
Response surface plots and contour plots show the effect of (**A**) extraction time and ultrasonic power, (**B**) extraction time and ethanol concentration, and (**C**) ultrasonic power and ethanol concentration on response of the total biflavonoids from *Selaginella doederleinii*.

The RSM 3D plot reflects the interaction between multiple variables. The steeper the slope of the response surface, the more sensitive the corresponding response values are to the conditions of interest, and the greater the influence of the factors on the SDTB extraction rate. Conversely, the flatter the slope of the response surface, the more insensitive the corresponding response values are to the conditions of interest, and the less influence the factors have on the SDTB extraction rate. As shown in Figure 4, all surfaces are convex downward and exhibit a maximum point, indicating that the effect of the interaction of the two variables on the SDTB extraction rate first increases and then decreases, and the SDTB extraction rate reaches the maximum value. The optimum extraction conditions obtained by RSM optimization were an extraction time of 41.74 min, extraction power of 307.99 W and ethanol concentration of 80.12%. At this time, the theoretically obtained SDTB yield was 22.30 mg/g. The extraction conditions were changed to 42 min, 320 W of power and an 80% ethanol concentration to consideration of the actual scenario. The yield of SDTB obtained under this condition was 22.26 ± 0.35 mg/g.

### 3.2. Effect of Purification Conditions

#### 3.2.1. Effect of Different Fillers

**Figure 5:**
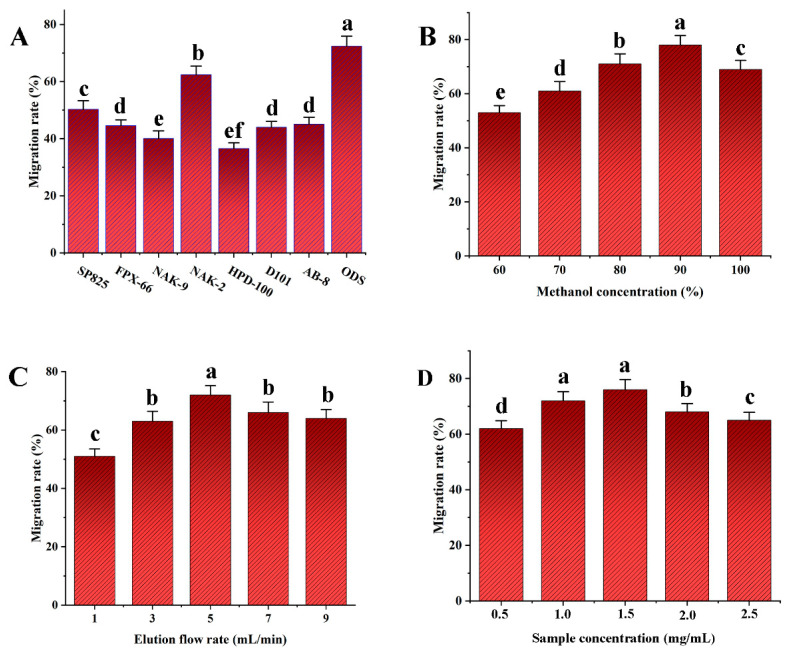
Transfer rates of different fillers (**A**); Transfer rates of biflavonoids in *Selaginella doederleinii* under different elution conditions (methanol concentration (**B**), elution flow rate (**C**), sample concentration (**D**)).

The transfer rate of NAK-2 resin was notably higher than that of the other resins, as Figure 5A illustrates. Additionally, ODS has a faster transfer rate and is more effective at enriching SDTB when compared to different macroporous resins. Therefore, ODS was selected as the best elution packing material to enrich SDTB. 

#### 3.2.2. Selection of Methanol Concentration

As shown in Figure 5B, the transfer rate of SDTB increased with increasing methanol concentration. The transfer rate declined somewhat at pure methanol and reached its maximum at 90% methanol elution. This may be due to the fact that as the methanol concentration gradually increases, it is favorable for methanol to promote the dissociation of SDTB from ODS through hydrogen-bonding interactions. Thus, 90% methanol was determined to be the ideal elution solvent. 

#### 3.2.3. Selection of Elution Flow Rate

As can be seen in Figure 5C, when the elution flow rate was increased from 1 mL/min to 5 mL/min, the SDTB transfer rate showed an increasing trend. At an elution flow rate of 5 mL/min, the SDTB transfer rate peaked. The transfer rate of SDTB then dropped when the elution flow rate increased to 9 mL/min. This may be due a flow rate that is too fast would flush out the impurities together, resulting in a lower content of SDTB being eluted. Therefore, the optimum elution flow rate was set at 5 mL/min. 

#### 3.2.4. Selection of Sample Concentration

As shown in Figure 5D, the SDTB transfer rate showed an increasing and then decreasing trend over the sample concentration range of 0.5 mg/mL to 2.5 mg/mL. The maximum SDTB transfer rate was observed at a sample concentration of 1.5 mg/mL. This may be due to the fact that a sample concentration that is too high can lead to the overloading of the ODS, which affects the elution content of the biflavonoids. Therefore, 1.5 mg/mL is the optimal sample concentration.

The optimum enrichment conditions for SDTB as described above were used for experimental validation. In other words, ODS was selected as the packing material, 90% methanol as the eluent, and 5 mL/min as the elution flow rate, and a sample concentration was 1.5 mg/mL. The transfer rate of SDTB obtained under this condition was 82.12 ± 3.48%.

Reversed-phase silica gel C18 is a solid-phase extraction material with hydrophobicity, which can effectively separate substances with different polarities. Its advantages include easy operation, good separation efficiency, and broad application, and it has been widely used for the separation of target components in medicinal herbs. Mirzahosseini et al. [59] isolated the ethanolic extract of *Centaurea bruguierana* using ODS and obtained three highly purified flavonoids (cirsimaritin, cirsilinelol and eupatilin). The MTT assay was used to confirm the low cytotoxicity of the three components in K562, AGS, MCF-7 and SW742 cell lines. Using ODS column chromatography, Lou et al. [60] extracted four known chemicals and two novel ones, armimelleolides A and B, from *Armillaria gallica*. With IC_50_ values ranging from 2.57 to 19.94 μM, all six compounds had noteworthy inhibitory effects on A549, HCT-116, M231 and W256 tumor cells.

The main components in SD extracts are biflavonoids, and there are few reports on SDTB enrichment and purification. In this study, the purification conditions were mainly examined in terms of the transfer rate of SDTB, and the transfer rate of SDTB reached 82.12 ± 3.48% after optimizing the conditions. The high transfer rate indicated a lower loss of biflavonoids during the purification process, which is essential for the enrichment of trace components in the herb. 

### 3.3. Validation of the Methodology

Based on the chromatographic peaks of the standards and samples, four biflavonoids including AMF, ROF, HIF and HEF were identified as the main active components of SD. The quantitative analysis of the above compounds was investigated under optimal conditions to accurately reflect the quality of the SD extract. The standard solutions of biflavonoids were used as indicators to investigate the linearity of the calibration curve. The standard curves were measured to explore the linear regression.

The standard curves of AMF, ROF, HIF and HEF were Y_AMF_ = 34861261X − 33697, Y_ROF_ = 29882101X + 21698, Y_HIF_ = 35384525X − 19170 and Y_HEF_ = 34681114X − 3119, respectively. Table 7 shows that the method was linear in the range of 2.00–120.00 μg/mL with an R^2^ greater than 0.9992. In addition, the limit of detection (LOD) and limit of quantification (LOQ) for the four biflavonoids were 0.118–0.615 μg/mL and 0.352–1.904 μg/mL, respectively.

The same sample was tested six times on the same day to calculate the intraday precision, and the interday precision was calculated for six consecutive days. Table 7 shows that the relative standard deviations (RSDs) of the intraday precision and interday precision were less than 2%, which indicats that the new method has good precision. The stability of the samples was tested at 25 °C for 0, 1, 2, 4, 8, 12 and 24 h. The results showed that the RSD values were less than 2%, indicating that the four biflavonoids in the SD extracts had a good stability over 24 h. The RSD values were in the range of 0.51–1.16% indicating the good repeatability of the method. The recoveries were in the range of 0.89–1.58%. In conclusion, the method is suitable for the determination of major biflavonoids in SD.

### 3.4. Enzyme Inhibitory Activity of SDTB Purifications

#### 3.4.1. AG Inhibitory Activity

**Figure 6:**
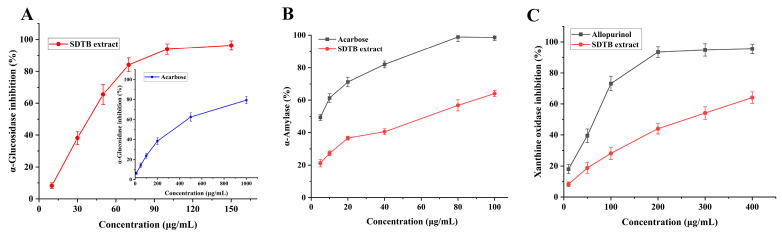
Enzyme activity results ((**A**) for α-glucosidase, (**B**) for α-amylase, (**C**) for xanthine oxidase (XOD)).

In recent years, the incidence of diabetes mellitus has increased dramatically worldwide [61]. Serious side effects, including hyperglycemia, diabetic ketoacidosis, cardiovascular disease and chronic renal failure, can result from delayed treatment [62]. Regulating blood glucose levels is a crucial and effective strategy for controlling diabetes. One of the ways to combat these diseases is to inhibit AG and α-amylase, as they play a key role in the development of these diseases. Additionally, AG is a key enzyme in glucose absorption in the gastrointestinal tract and inhibition of its activity prevents postprandial hyperglycemia [63,64]. α-Glucosidase and α-amylase are two promising targets for the treatment of T2DM. The results of the current investigation, which assessed the inhibitory activity of the SD extract on AG, are displayed in Figure 6A. At 150 μg/mL, the SD extract inhibited AG by 96.26 ± 2.83%; its IC_50_ value was 57.46 μg/mL, lower than the IC_50_ value of the control drug, acarbose (374.58 μg/mL). This suggests that SD extract has a promising antidiabetic effect.

#### 3.4.2. α-Amylase Inhibitory Activity

α-Amylase is a key enzyme in the hydrolysis of long carbohydrates into short glucans for intracellular transport. Inhibition of α-amylase activity delays carbohydrate digestion in the small intestine and reduces postprandial blood glucose levels [65]. Flavonoids are a ubiquitous naturally occurring class of compounds that have long been recognized as active in many systems with multiple benefits for human health [66,67,68]. They might therefore be a safe and well-tolerated treatment for T2DM. In this study, the inhibitory activity of SD extract on α-amylase was evaluated and the results are shown in Figure 6B. The inhibition of α-amylase by SD extract at a concentration of 100 μg/mL was 64.02 ± 1.86%, and its IC_50_ value was 50.85 μg/mL, which was higher than that of the positive control, acarbose (6.34 μg/mL). However, its concentration was lower and had some hypoglycemic efficacy. SD extract can be used as a potentially effective drug to inhibit both α-glucosidase and α-amylase for better glycemic maintenance results while minimizing side effects.

#### 3.4.3. XOD Inhibitory Activity

Uric acid is a substance formed when the body breaks down purines in the blood. Excess uric acid (hyperuricemia) causes chronic inflammatory arthritis leading to gout. Currently, the two primary medications used for uric acid-lowering therapy are diuretics, which enhance urea excretion, and XOD inhibitors, which decrease uric acid synthesis [69]. The results of the current investigation, which assessed the inhibitory activity of SD extract on XOD, are displayed in Figure 6C. At 400 μg/mL, SD extract inhibited XOD by 64.05 ± 3.73%; its IC_50_ value was 289.67 μg/mL, which was higher than the IC_50_ value of the control drug, allopurinol (57.49 μg/mL). Its concentration was lower, though, and it does have some ability to fight gout.

With the abundance of dietary products, especially high-sugar and high-purine foods, the number of people suffering from diabetes and gout has been on the rise in recent years. The hypoglycemic and antigout effects of biflavonoids have received widespread attention. Human islet amyloid polypeptide (hIAPP) is synthesized in pancreatic β-cells and co-secreted with insulin, and its amyloid deposition aggravates type II diabetes mellitus (T2DM). According to research by Xu et al. [70], AMF and bilobetin have the ability to prevent hIAPP from aggregating and to interfere with its fibrillation. This can lessen peptide oligomerization and increase the vitality of INS-1 cells, both of which may have therapeutic benefits for T2DM. The strong AG inhibitory activity of ginkgetin, AMF and sciadopitysin was determined by Wu et al. [71] with IC_50_ values of 1.79 μM, 3.28 μM and 8.29 μM, respectively. The order of enzyme inhibitory activity of the different constituents of Ginkgo biloba was also verified to be biflavone > flavone > flavone glycoside > flavone biglycoside. Zhang et al. [72] found that AMF was able to downregulate the levels of NO, TNF-α and lactate dehydrogenase (LDH); downregulate the mRNA expression of IL-1β, TNF-α, cystatinase-1 and NLRP3 in an acute gout mouse model; and alleviate gout arthritis by inhibiting the NLRP3/ASC/Caspase-1 axis. In the present study, the AG and XOD inhibitory activities of SD extract were determined. In comparison to the positive control, the extract exhibited superior enzyme inhibitory activity, according to the results. This was associated with the higher content of biflavonoid constituents in the extract. Therefore, more research on the enzyme inhibitory capabilities of the specific biflavonoid ingredients is required for comprehensive analyses that will find novel medications to treat gout and diabetes.

### 3.5. Molecular Docking

The docking binding energies are shown in Table 8. Acarbose, AMF and ROF were the three small molecules whose binding energies to AG were −7.810 kcal/mol, −10.40 kcal/mol and −11.33 kcal/mol, respectively. The binding energies of the small molecules allopurinol, AMF and ROF to XOD were −6.338 kcal/mol, −9.962 kcal/mol and −10.21 kcal/mol, respectively. The docking simulations suggest that the small molecules AMF and ROF may have the potential to interact with the binding pocket of AG, but further experimental validation is needed to confirm their inhibitory effects.

**Figure 7:**
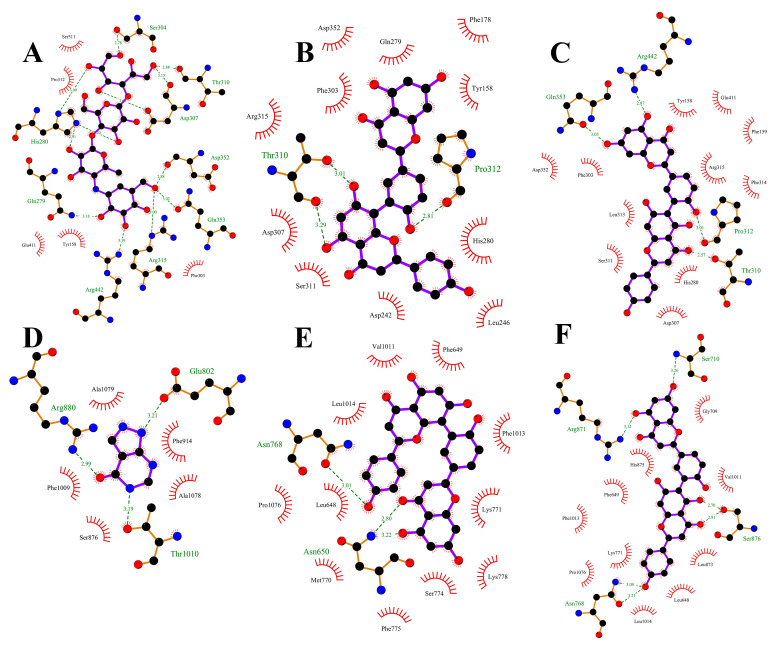
Two-dimensional plots of the binding details of α-glucosidase protein and the small molecules acarbose (**A**), AMF (**B**) and ROF (**C**), and 2D plots of the binding details of XOD protein and the small molecules allopurinol (**D**), AMF (**E**) and ROF (**F**). Dashed lines indicate hydrogen bonds and red eyelashes indicate hydrophobic interaction amino acids.

**Figure 8:**
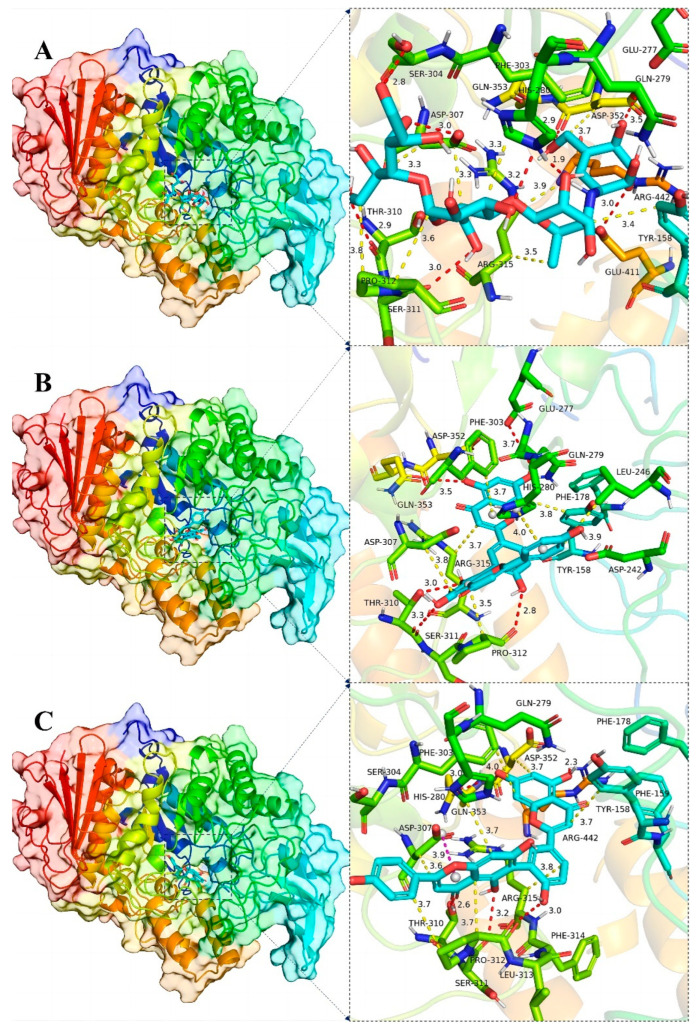
Global view (**left**) of the binding of α-glucosidase protein and the small molecules acarbose (**A**), AMF (**B**) and ROF (**C**), and 3D view of the binding details of the optimal structure (**right**). Proteins are represented as cartoons and small molecules as stick models; red dashed lines indicate hydrogen bonds, blue dashed lines indicate π–π interactions, yellow dashed lines indicate van der Waals/hydrophobic interactions and pink dashed lines indicate ion–π interactions.

Plots demonstrating the interactions between AG proteins and the small molecules ROF, AMF and acarbose are displayed in Figure 7A–C and Figure 8. GLN353 is an important amino acid that forms hydrogen bonds with the small molecules acarbose and ROF. Important amino acids that also form hydrogen bonds with small molecules are THR310, PRO312 and ARG442. Thr158, PHE303 and SER311 are also common residues involved in van der Waals interactions. Hydrogen bonding plays a major role in the binding of all small molecules to AG, and numerous residues involved in van der Waals interactions also play an important role. The ionic π interaction with ASP307 in ROF binding is also crucial for maintaining the binding.

**Figure 9:**
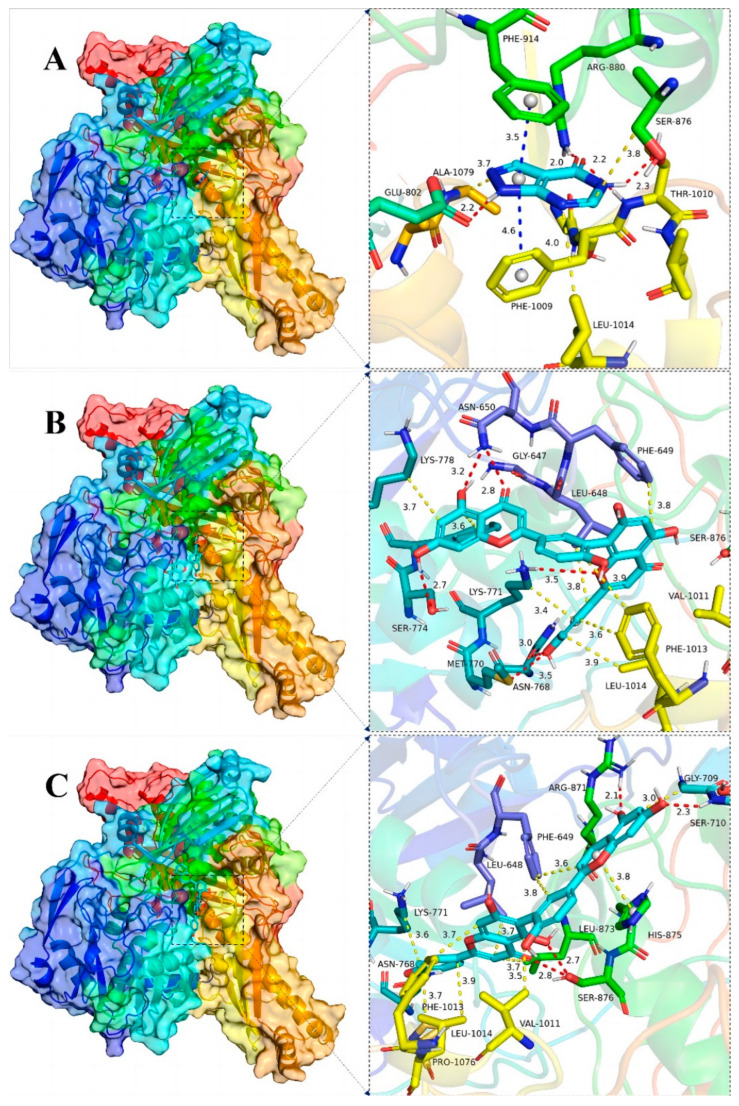
Global view (**left**) of the binding of the XOD proteins and the small molecules allopurinol (**A**), AMF (**B**) and ROF (**C**) as well as 3D view of the binding details of the optimal structures (**right**). Proteins are represented as cartoons and small molecules as stick models; red dashed lines indicate hydrogen bonding, blue dashed lines indicate π–π interactions and yellow dashed lines indicate van der Waals/hydrophobic interactions.

The binding details were then analyzed to map the interactions of the XOD protein with the small molecules allopurinol, AMF and ROF as shown in Figure 7D–F and Figure 9. Allopurinol is consistent with its primary role in the literature [73,74], whereas for AMF as well as ROF it binds predominantly to the outer surface region of the structural domain of the allopurinol binding site. This may be due to the presence of a stronger rigid region for the two molecules, being unable to penetrate deeper into this pocket. ASN768 is an important residue involved in hydrogen bonding and LEU648, LYS771, PHE1013 and PRO1076 are important residues involved in van der Waals interactions. In the binding of all small molecules to XOD, hydrogen bonding plays a major role, and numerous residues involved in van der Waals interactions also play important roles.

To summarize, XOD is bound by the small molecules allopurinol, AMF and ROF, while AG is bound by the small molecules acarbose, AMF and ROF. The following is the binding capacity ranking, from strongest to weakest: AG for ROF > AMF > acarbose; XOD for ROF > AMF > allopurinol. 

### 3.6. Molecular Dynamic

**Figure 10:**
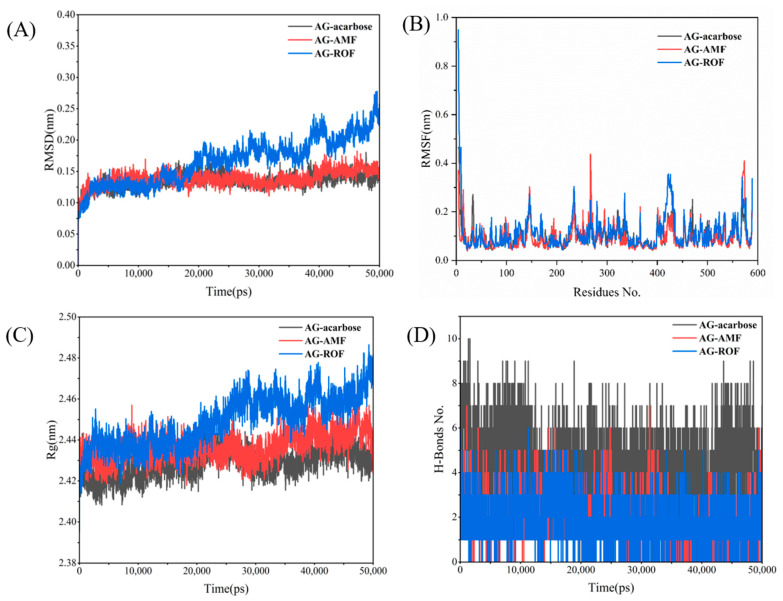
AG MD results: (**A**) RMSD; (**B**) RMSF; (**C**) Rg; (**D**) H-bonds.

To further investigate the interactions between the receptor protein and small molecules during motion, and to assess the stability of the binding site, we conducted a 50 ns molecular dynamics (MD) simulation of the complex to verify the system’s dynamic stability. The RMSD of the AG complex system is shown in Figure 10A. After approximately 5 ns of MD simulation, the system began to stabilize. Additionally, Figure 10A shows that the RMSD of the AG–ROF complex gradually increases after 20 ns, indicating that the active site of the protein undergoes changes, allowing the ROF molecule to better fit into the protein pocket. Figure 10B illustrates the fluctuations of individual residues in the three AG complexes. In general, the AG–AMF and AG–ROF complexes exhibit greater residue fluctuations, especially in certain regions (such as residues 300 to 400), indicating higher flexibility in these areas. Figure 10C shows the variation in the radius of gyration (Rg) over time for the three AG complexes. The AG–acarbose complex exhibits the most stable Rg value, maintaining around 2.42 nm, indicating that the complex retains a tight overall structure. The Rg value of AG–AMF is slightly higher than that of AG–acarbose, while the AG–ROF complex exhibits larger fluctuations, with an upward trend in the later stages of the simulation, suggesting that the structure of the AG–ROF complex becomes more relaxed over time. Figure 10D shows the changes in the number of hydrogen bonds in the three AG complexes. The AG–acarbose complex has the most stable hydrogen bonds (approximately 4–8), indicating strong interactions between acarbose and the protein. The AG–AMF complex has fewer hydrogen bonds, with greater fluctuations, while the AG–ROF complex has the fewest hydrogen bonds and shows the least stability, indicating weaker interactions between ROF and the protein.

**Figure 11:**
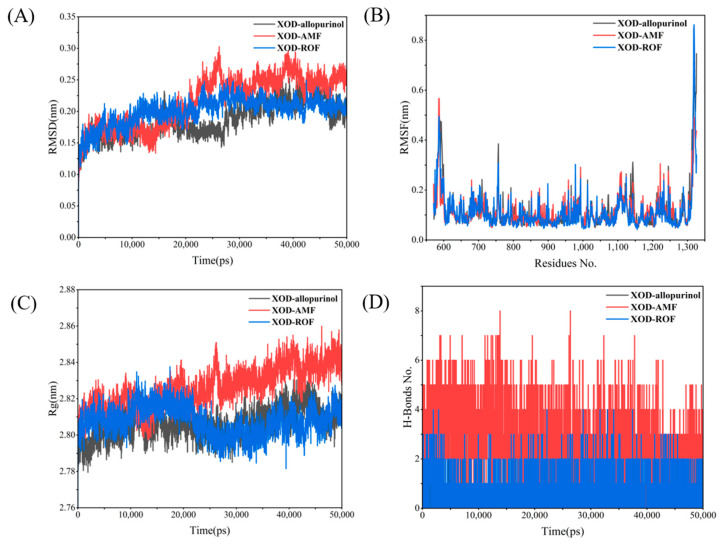
XOD MD results: (**A**) RMSD; (**B**) RMSF; (**C**) Rg; (**D**) H-bonds.

By monitoring the stability of allopurinol in the active site, snapshots were extracted at different times during the simulation as shown in Figure S1, where allopurinol remained in the active pocket throughout the simulation, indicating that allopurinol was able to bind well to the protein XOD. Figure 11A presents the RMSD changes for the XOD complexes with the three ligands. The RMSD of the XOD–allopurinol complex remains between 0.15 and 0.2 nm throughout the simulation, with minimal fluctuations, indicating a stable structure. The XOD–AMF complex shows a significant increase in RMSD in the later stages of the simulation, indicating substantial conformational changes. The XOD–ROF complex remains relatively stable for most of the simulation. Figure 11B shows that the XOD–AMF and XOD–ROF complexes exhibit significant residue fluctuations, especially towards the C-terminal region (around residue 1300), indicating greater flexibility. In contrast, the XOD–allopurinol complex shows smaller residue fluctuations, reflecting a higher local stability. Figure 11C demonstrates that the Rg value of the XOD–allopurinol complex remains around 2.80 nm with minimal fluctuations, indicating a compact and stable structure. The Rg value of the XOD–AMF complex is significantly higher and more variable, especially towards the end of the simulation, suggesting that the structure becomes increasingly relaxed. The Rg value of the XOD–ROF complex lies between the other two, showing moderate compactness. Finally, Figure 11D illustrates the changes in hydrogen bond numbers over time, reflecting the interaction strength of the complexes. The XOD–AMF complex has the most hydrogen bonds and is relatively stable, indicating strong interactions between AMF and XOD. The XOD–allopurinol complex has fewer hydrogen bonds but maintains reasonable stability. The XOD–ROF complex has the fewest hydrogen bonds, with noticeable fluctuations, suggesting weaker interactions between ROF and XOD.

MM-PBSA (Molecular Mechanics Poisson–Boltzmann Surface Area) is highly popular for predicting binding free energy (ΔG_bind) because it tends to be more accurate than most scoring functions used in molecular docking. It provides the relative free binding energy of a ligand forming a complex with a protein receptor, as shown in Table 9. In our results, the binding free energy for the AG–ROF system (ΔG = −53.341 kcal/mol) was greater than that of the AG–AMF system (ΔG = −42.00 kcal/mol), which in turn was greater than the AG–acarbose system (ΔG = −27.21 kcal/mol). Similarly, for the XOD complexes, the binding free energy of XOD–ROF (ΔG = −81.37 kcal/mol) was greater than that of XOD–AMF (ΔG = −54.65 kcal/mol), which was greater than the XOD–acarbose system (ΔG = −16.39 kcal/mol). These results suggest that at different active sites, the binding affinity of the ligands to the target receptors increases, indicating the higher stability of the complexes. This may be due to the small molecules altering the original structure at different binding sites, leading to the formation of more stable conformations when the ligands bind to the target proteins.

## 4. Conclusions

In this work, the key parameters required for the extraction of SD extract were obtained by single-factor combinatorial RSM optimization. Meanwhile, the SD extract purification process was effectively constructed, which provides a model for the promotion and application of SD extract in the future. In addition, the potential antidiabetic and gouty effects of the SD extract were verified. The extract was primarily dominated by biflavonoid components, and the experimental results were supported by molecular docking and molecular dynamics simulation data. Therefore, further improvement of the purity of the SD extract could enhance the developmental value of its pharmaceutical constituents.

## Figures and Tables

**Table 1:** Box–Behnken factor level design table.

Coding Factors	Unit	Coding Levels		
		−1	0	1
A: extraction time	min	30	40	50
B: extraction power	W	280	320	360
C: ethanol concentration	%	60	70	80

**Table 2:** Process for measuring α-glucosidase (AG) activity. (PBS, phosphate buffer solution; pNPG, p-nitrophenyl-α-d-glucopyranoside).

Reagent	Control Group	Sample Group	Sample Blank Group
PBS buffer solution (0.1 mol/L)	100 µL	80 µL	105 µL
α-glucosidase (0.2 U/mL)	25 µL	25 µL	\
sample solution	\	20 µL	20 µL
Incubate at 37 °C for 20 min			
pNPG (4 mM)	25 µL	25 µL	25 µL
Incubate at 37 °C for 15 min			
Na_2_CO_3_ (0.2 mol/L)	50 µL	50 µL	50 µL
Measurement of absorbance at 405 nm			

**Table 3:** Process for measuring α-amylase activity.

Reagent	Control Group	Sample Group	Sample Blank Group
sample solution	\	500 µL	500 µL
α-amylase (0.1 mg/mL)	500 µL	500 µL	\
PBS buffer solution (0.1 M)	500 µL	\	500 µL
Incubate at 37 °C for 10 min			
1% starch solution	500 µL	500 µL	500 µL
Incubate at 37 °C for 10 min			
DNS reagent	1 mL	1 mL	1 mL
Boiled for 5 min, cooled and diluted with 10 mL of distilled water and finally the absorbance was measured at 540 nm			

**Table 4:** Determination process of xanthine oxidase (XOD) activity.

Reagent	Control Group	Control Blank Group	Sample Group	Sample Blank Group
PBS buffer solution (0.2 M, pH = 7.5)	140 µL	160	120 µL	140 µL
XOD (0.1 U/mL)	20 µL	\	20 µL	\
sample solution	\	\	20 µL	20 µL
Incubate at 37 °C for 10 min				
xanthine (2 mM, pH = 7.5)	20 µL	20 µL	20 µL	20 µL
Incubate at 37 °C for 30 min				
HCl (1 M)	40 µL	40 µL	40 µL	40 µL
Measurement of absorbance at 290 nm				

**Table 5:** Box–Behnken experimental results.

No.	A (min)	B (W)	C (%)	Y (mg/g)
1	30	320	70	17.62
2	40	360	70	16.44
3	40	280	90	18.36
4	30	280	80	20.60
5	40	320	80	22.07
6	50	320	70	19.01
7	50	360	80	18.93
8	30	360	80	19.39
9	40	320	80	22.20
10	40	320	80	22.45
11	30	320	90	19.18
12	40	280	70	19.85
13	40	360	90	18.78
14	40	320	80	22.28
15	50	280	80	21.01
16	50	320	90	19.69
17	40	320	80	21.81

**Table 6:** Analysis of variance for the Y regression equation.

Source	Sum of Squares	df	*F* Value	*p* Value	*R* ^2^	*R*^2^ (Adj)	Significant
Model	49.81	9	40.19	<0.0001	0.9810	0.9566	significant
A	0.419	1	3.04	0.1247			
B	4.92	1	35.73	0.0006			
C	1.19	1	8.65	0.0217			
AB	0.1876	1	1.36	0.2813			
AC	0.1939	1	1.41	0.2741			
BC	3.67	1	26.67	0.0013			
A^2^	2.91	1	21.14	0.0025			
B^2^	7.66	1	55.65	0.0001			
C^2^	25.39	1	184.37	<0.0001			
Residual	0.964	7					
Lack of Fit	0.7363	3	4.31	0.096			not significant
Pure Error	0.2278	4					
Cor Total	50.78	16					

**Table 7:** Method validation for four biflavonoids.

Analyte	Calibration Curve	R^2^	LOD (μg/mL)	LOQ (μg/mL)	Intraday Precision (*n* = 6) ^a^	Interday Precision (*n* = 6) ^a^	Stability (*n* = 7) ^a^	Repeatability (*n* = 6) ^a^	Recovery (*n* = 9) ^a^
AMF	y = 34861261X − 33697	0.9992	0.535	1.567	1.17	1.32	0.72	0.51	0.89
ROF	y = 29882101X + 21698	0.9994	0.615	1.904	0.89	1.15	1.88	1.14	1.03
HIF	y = 35384525X − 19170	0.9992	0.279	0.862	1.05	1.47	1.48	1.01	1.52
HEF	y = 34681114X − 3119	0.9995	0.118	0.352	1.06	1.14	1.12	1.16	1.58

LOD, limit of detection (S/N = 3); LOQ, limit of quantification (S/N = 10). ^a^ Intraday precision, interday precision, stability, repeatability and recovery are expressed as the RSD (%) of the peak area; AMF, Amentoflavone; ROF, Robustaflavone; HIF, Hinokiflavone; HEF, Heveaflavone.

**Table 8:** Binding energies of small molecules to AG and XOD proteins.

Proteins	Small Molecules	Binding Energies (kcal/mol)
AG	acarbose	−7.810
	AMF	−10.40
	ROF	−11.33
XOD	allopurinol	−6.338
	AMF	−9.962
	ROF	−10.21

**Table 9:** Binding free energies of ligands and the different energy contributions.

No.	ΔEVDW (kcal/mol)	ΔEEEL (kcal/mol)	ΔGGB (kcal/mol)	ΔGEURF (kcal/mol)	ΔGbind (kcal/mol)
AG–acarbose	−69.24	−382.16	432.55	−8.35	−27.21
AG–AMF	−57.38	−16.73	38.54	−6.44	−42.00
AG–ROF	−71.53	−18.13	43.96	−7.64	−53.34
XOD–allopurinol	−20.76	−0.92	7.94	−2.64	−16.39
XOD–AMF	−80.03	−7.15	39.98	−7.45	−54.65
XOD–ROF	−95.08	−12.93	34.88	−9.24	−81.37

## Data Availability

Data are contained within the article.

## Supplementary Materials

The following supporting information can be downloaded at https://www.mdpi.com/article/10.3390/antiox13101184/s1, Figure S1. Allopurinol snapshots at different time periods during the redynamic simulation.
