# Supplementary figures and images for: Antidiabetic and Antigout Properties of the Ultrasound-Assisted Extraction of Total Biflavonoids from Selaginella doederleinii Revealed by In Vitro and In Silico Studies
Source: Antioxidants (Basel). 2024 Sep 30;13(10):1184. doi: 10.3390/antiox13101184 (PMC11504096; doi:10.3390/antiox13101184)

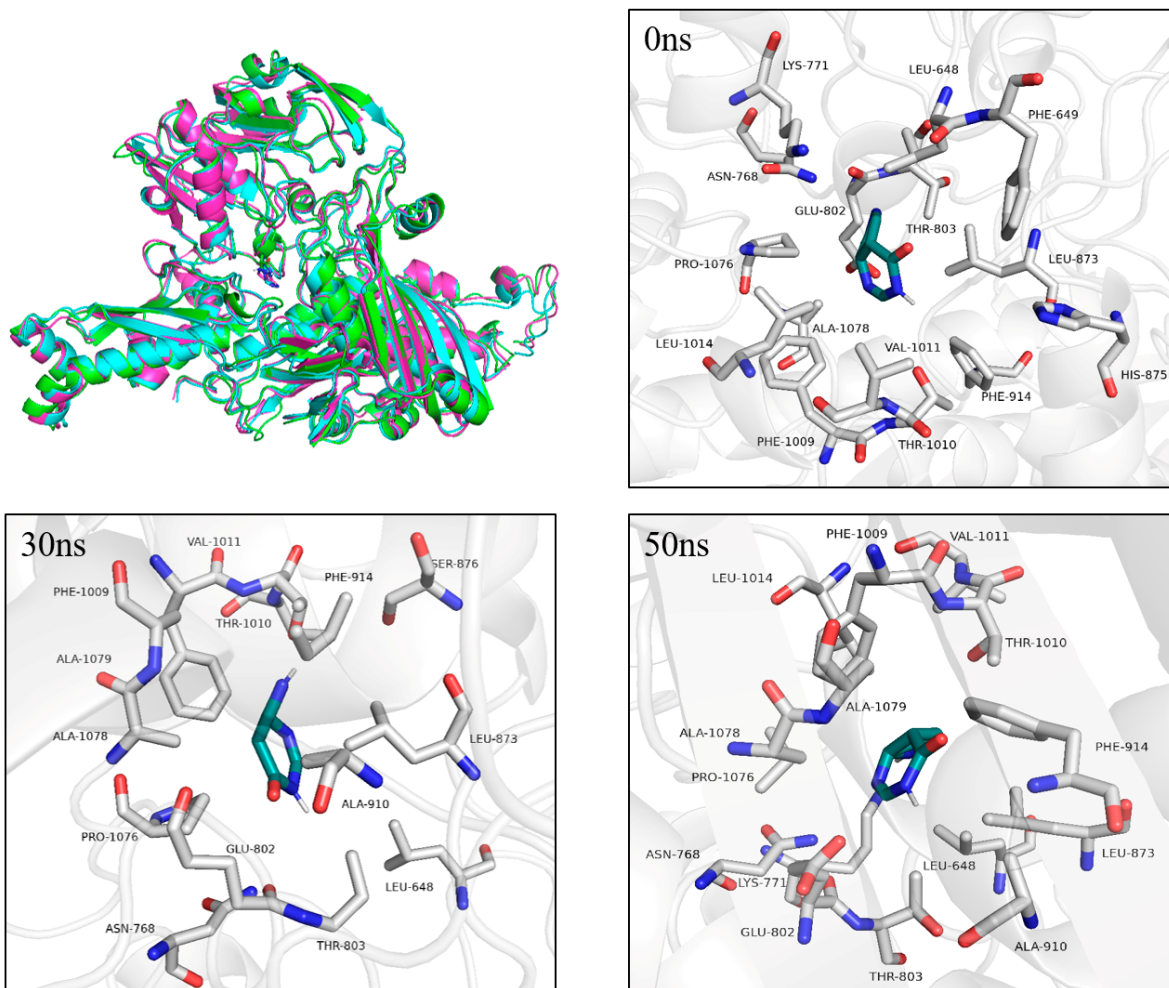

**Figure S1.** Allopurinol snapshots at different time periods during the redynamic simulation.

Supplement: Supplementary file 1 [file antioxidants-13-01184-s001.zip › antioxidants-3204864-supplementary.pdf]
